# Hypoxia‐Enhanced Wharton's Jelly Mesenchymal Stem Cell Therapy for Liver Fibrosis: A Comparative Study in a Rat Model

**DOI:** 10.1002/kjm2.70053

**Published:** 2025-06-30

**Authors:** Wei‐Ting Kuo, Chen‐Yuan Hsiao, Sheng‐Hao Chiu, Shu‐Cheng Chou, Ching‐Shu Chiang, Jui‐Yu Chen, Solomon Chih‐Cheng Chen, Tien‐Hua Chen, Jia‐Fwu Shyu, Chi‐Hung Lin, Pei‐Jiun Tsai

**Affiliations:** ^1^ Institute of Clinical Medicine National Yang Ming Chiao Tung University Taipei Taiwan; ^2^ Department of Surgery Chiayi Christian Hospital Chiayi Taiwan; ^3^ Division of Cardiovascular Surgery, Department of Surgery Taipei Medical University Hospital Taipei Taiwan; ^4^ Department of Surgery, School of Medicine, College of Medicine Taipei Medical University Taipei Taiwan; ^5^ Taipei Heart Institute Taipei Medical University Taipei Taiwan; ^6^ Department of Biology and Anatomy National Defense Medical Center Taipei Taiwan; ^7^ Institute of Anatomy and Cell Biology National Yang Ming Chiao Tung University Taipei Taiwan; ^8^ Division of General Surgery, Department of Surgery Taipei Veterans General Hospital Taipei Taiwan; ^9^ Graduate Institute of Medical Sciences National Defense Medical Center Taipei Taiwan; ^10^ Department of Pediatrics Chiayi Christian Hospital Chiayi Taiwan; ^11^ Institute of Microbiology and Immunology National Yang Ming Chiao Tung University Taipei Taiwan; ^12^ Department of Biological Science and Technology National Yang Ming Chiao Tung University Hsinchu Taiwan; ^13^ Cancer Progression Research Center National Yang Ming Chiao Tung University Taipei Taiwan; ^14^ Department of Critical Care Medicine Taipei Veterans General Hospital Taipei Taiwan; ^15^ Trauma Center, Department of Surgery Taipei Veterans General Hospital Taipei Taiwan

**Keywords:** fibrosis regeneration, hypoxia preconditioning, liver fibrosis, stem cell therapy, Wharton's jelly mesenchymal stem cells (WJ‐MSCs)

## Abstract

Liver fibrosis is a progressive disease that can lead to cirrhosis and liver failure, with limited treatment options. Wharton's jelly‐derived MSCs (WJ‐MSCs) have immunomodulatory and antifibrotic potential. Hypoxia preconditioning enhances MSC survival and paracrine activity, but its effects in liver fibrosis remain unclear. This study compares hypoxia and normoxia WJ‐MSCs in a CCl_4_‐induced liver fibrosis rat model. Sprague–Dawley rats received chronic CCl_4_ to induce fibrosis. At Week 8, normoxia or hypoxia WJ‐MSCs were injected via the portal vein. Liver function was assessed using biochemical markers (ALT, AST, T‐Bil, albumin), PET/MR imaging, and qPCR for IL‐1β and IL‐6. Fibrosis regression was evaluated via ultrasound, histology, and collagen quantification. Regeneration was analyzed through Ki67 immunostaining and qPCR for Ki67 and HGF. MSC engraftment was determined by hNA immunohistochemistry. Both normoxia and hypoxia WJ‐MSCs improved liver function, with hypoxia WJ‐MSCs showing greater AST and T‐Bil reductions. PET/MR imaging demonstrated superior metabolic recovery in the hypoxia group, with greater ^18^F‐FDG uptake reduction. Histological analysis confirmed more significant fibrosis regression and collagen reduction in the hypoxia group. Gene expression analysis showed stronger suppression of TGF‐β, α‐SMA, and collagen I. Liver regeneration markers Ki67 and HGF were significantly upregulated with a greater HGF increase in the hypoxia group. Additionally, hypoxia WJ‐MSCs exhibited higher engraftment and reduced pulmonary entrapment, indicating improved liver homing. Both normoxia and hypoxia WJ‐MSCs improved liver fibrosis, but hypoxia preconditioning further enhanced liver function, fibrosis regression, and metabolic recovery, supporting its therapeutic superiority.

## 1 Introduction

Liver fibrosis is a progressive condition marked by excessive extracellular matrix (ECM) deposition due to chronic liver injury from viral infections, alcohol abuse, metabolic disorders, and nonalcoholic fatty liver disease (NAFLD). If untreated, it can progress to cirrhosis and hepatocellular carcinoma (HCC), increasing global morbidity and mortality [1, 2, 3]. Current treatments, including pharmacological therapy and liver transplantation, are limited by poor efficacy, high costs, and donor shortages. Therefore, novel regenerative therapies are urgently needed to halt fibrosis progression and promote liver repair.

The carbon tetrachloride (CCl_4_)‐induced liver fibrosis rat model is widely used for its reproducibility and similarity to human fibrosis [4, 5]. CCl_4_ exposure triggers oxidative stress, hepatocyte damage, and hepatic stellate cell (HSC) activation, leading to excessive ECM deposition and fibrosis [6]. This model allows quantitative evaluation of therapeutic efficacy using biochemical markers alanine transaminase (ALT), alanine aminotransferase (AST), and histological scoring. Despite species differences, it remains a reliable preclinical platform for testing antifibrotic therapies before clinical application [7].

Mesenchymal stem cells (MSCs) are a promising therapy for liver fibrosis [8, 9], with preclinical and early clinical studies showing therapeutic potential. Among MSC sources, Wharton's jelly‐derived MSCs (WJ‐MSCs) offer key advantages, including low immunogenicity, high proliferative capacity, and ethical accessibility [10, 11].

Derived from umbilical cords, WJ‐MSCs eliminate donor morbidity and allow transplantation without strict human leukocyte antigen (HLA) matching. They secrete anti‐inflammatory cytokines (IL‐10, TGF‐β), suppressing immune activation and inflammation while maintaining multipotency, differentiating into osteogenic, chondrogenic, adipogenic, hepatogenic, and neurogenic lineages [12, 13]. Their resistance to oxidative stress and apoptosis, combined with genomic stability in extended culture, reduces tumorigenicity risks, making them a strong candidate for liver fibrosis treatment [14].

Hypoxia preconditioning (culturing MSCs in low‐oxygen conditions before transplantation) enhances their therapeutic potential by mimicking the hypoxic microenvironment of damaged liver tissue [15, 16]. This process upregulates hypoxia‐inducible factors (HIF‐1α, HIF‐2α), enhances anti‐inflammatory cytokine secretion, and activates pro‐survival signaling, improving MSC survival, migration, and paracrine function [17, 18, 19]. Additionally, hypoxia‐stimulated MSCs secrete vascular endothelial growth factor (VEGF) and hepatocyte growth factor (HGF), promoting angiogenesis and liver regeneration [20, 21]. They also upregulate matrix metalloproteinases (MMP‐2, MMP‐9), facilitating ECM remodeling and fibrosis reduction [13, 22]. While hypoxia‐preconditioned MSCs have shown superior efficacy in fibrosis models [7, 15], research on hypoxia WJ‐MSCs in liver fibrosis remains limited. This study compares the therapeutic effects of hypoxia‐ and normoxia‐preconditioned WJ‐MSCs to assess whether hypoxia preconditioning enhances antifibrotic efficacy.

We hypothesize that both WJ‐MSC groups will improve liver fibrosis, but hypoxia WJ‐MSCs will demonstrate greater antifibrotic, anti‐inflammatory, and regenerative effects. This is likely due to hypoxia‐induced upregulation of survival and paracrine signaling pathways, enhancing metabolic recovery, fibrosis resolution, and immune modulation. If confirmed, these findings could establish hypoxia preconditioning as an optimized strategy to enhance MSC‐based liver fibrosis therapy and support its clinical application.

## 2 Materials and Methods

### 2.1 Isolation and Culture of Human Wharton's Jelly‐Derived Mesenchymal Stem Cells (WJ‐MSCs)

Human umbilical cords were collected aseptically and stored at 4°C in Hank's Balanced Salt Solution (HBSS). Wharton's jelly was dissected, minced, and centrifuged at 250 g for 5 min before washing with serum‐free Dulbecco's Modified Eagle's Medium (DMEM). The pellet underwent enzymatic digestion with 1% collagenase type II (37°C, 18 h), followed by 0.025% trypsin (30 min). Isolated cells were cultured in mesenchymal stem cell medium (MSCM) under normoxic (21% O_2_) or hypoxic (1.5% O_2_) conditions at 37°C. The medium was replaced every 2–3 days, and cells were passaged at 80% confluence for transplantation.

### 2.2 Animal Model and Experimental Design

Male Sprague–Dawley rats (6 weeks old, ~200 g) were housed under controlled conditions (18°C–25°C, 40%–70% humidity, 12‐h light/dark cycle). Liver fibrosis was induced by twice‐weekly oral administration of 50% CCl_4_ in olive oil (1 mL/kg) for 8 weeks. Fibrosis progression was monitored via biochemical analysis and ultrasound. At Week 8, animals were randomized into four groups: healthy control, fibrosis control, normoxia WJ‐MSCs, and hypoxia WJ‐MSCs. Each treatment group received 1 × 10^7^ WJ‐MSCs via direct injection into the portal vein (PV). Post‐transplantation, animals were monitored for an additional 4 weeks, with blood samples collected at Weeks 2 and 4. At Week 12, all animals were euthanized for tissue analysis.

### 2.3 Stem Cell Transplantation

Rats were anesthetized with Zoletil (30 mg/kg) and xylazine (10 mg/kg, intraperitoneally) before laparotomy. After sterilization, the surgical area was shaved, sterilized with iodine, and an incision was made to expose the PV. A scalp vein needle was inserted into the PV to administer 2 mL of WJ‐MSC suspension at 0.6 mL/min, followed by a 0.5 mL saline flush. Hemostasis was ensured with sterile gauze, and incisions were sutured in layers. Postoperatively, rats received ketoprofen (3 mg/kg, intramuscularly) and Stazolin (50 mg/kg, intraperitoneally) before being returned to temperature‐controlled cages.

### 2.4 Physiological Monitoring and Imaging

Body weight, food intake, and water consumption were recorded weekly. Ultrasound imaging was performed every 4 weeks to assess fibrosis progression. ^18^F‐FDG PET/MR was conducted at Weeks 8 and 12 to evaluate metabolic activity and inflammation. Anesthetized rats received 1 mCi of ^18^F‐FDG via tail vein, followed by imaging 45 min later using a small‐animal PET/MR scanner. This approach provided quantitative insights into metabolic recovery and inflammatory modulation, enhancing the evaluation of WJ‐MSC therapy's impact on liver fibrosis.

### 2.5 Serum Biochemical Analysis

Blood samples were collected via the tail vein at Weeks 0, 2, 4, 6, 8, 10, and 12 using heparinized syringes (125 IU/mL). Serum was separated by centrifugation and analyzed using an automated chemistry analyzer (Fujifilm DRI‐CHEM NX600i) for AST, ALT, T‐BIL, albumin, and ammonia (NH_3_).

### 2.6 Histological and Immunohistochemical Analyses

At the end of the study, liver tissues were harvested, fixed in 10% neutral‐buffered formalin, embedded in paraffin, and sectioned for H&E (morphology), Masson's trichrome (collagen), and Sirius Red (fibrosis quantification) staining. For immunofluorescence, sections underwent antigen retrieval and blocking before overnight incubation. Primary antibodies against TGF‐β (1:200), α‐SMA (1:1000), Ki‐67 (1:50), and human nuclear antigen (hNA) (1:200) were incubated overnight at 4°C. After washing, sections were incubated with secondary antibodies for 1 h at room temperature. Nuclei were counterstained with FluoroQtest Mounting Medium containing DAPI, and slides were imaged using fluorescence microscopy.

### 2.7 Total Collagen Assay

Liver tissue homogenates (100 mg/mL) were mixed with 10 N NaOH, heated at 120°C for 1 h, and neutralized with 10 N HCl. The supernatant was centrifuged, and 2–10 μL aliquots were dried in a 96‐well plate at 65°C. Each well was treated with Oxidation Mix, incubated for 20 min, followed by Developer and DMAB Concentrate, then heated at 65°C for 45 min. Absorbance at 560 nm was measured using a microplate reader.

### 2.8 Quantitative Real‐Time PCR (qPCR)

Total RNA was extracted from liver and lung tissues using an RNA isolation kit, followed by cDNA synthesis via a reverse transcription kit. qPCR was performed using SYBR Green chemistry on a StepOnePlus system to quantify fibrosis markers (TGF‐β, α‐SMA, collagen I), inflammatory cytokines (IL‐1β, IL‐6), and regeneration markers (Ki67, HGF). GAPDH was used as the reference gene, and relative expression levels were calculated via quantification (threshold cycle, Ct) to compare the mRNA expression levels between groups. All reactions were conducted in triplicate.

### 2.9 Statistical Analysis

Data were analyzed using GraphPad Prism v10.3.1. Differences between groups were assessed using one‐way/two‐way ANOVA followed by Tukey's post hoc test or Student's *t*‐test, as appropriate. Image quantification was performed using ImageJ software. Results are presented as mean ± standard deviation, with *p* < 0.05 considered significant.

## 3 Results

### 3.1 Ultrasound Imaging Analysis of Liver Fibrosis Progression

**FIGURE 1:**
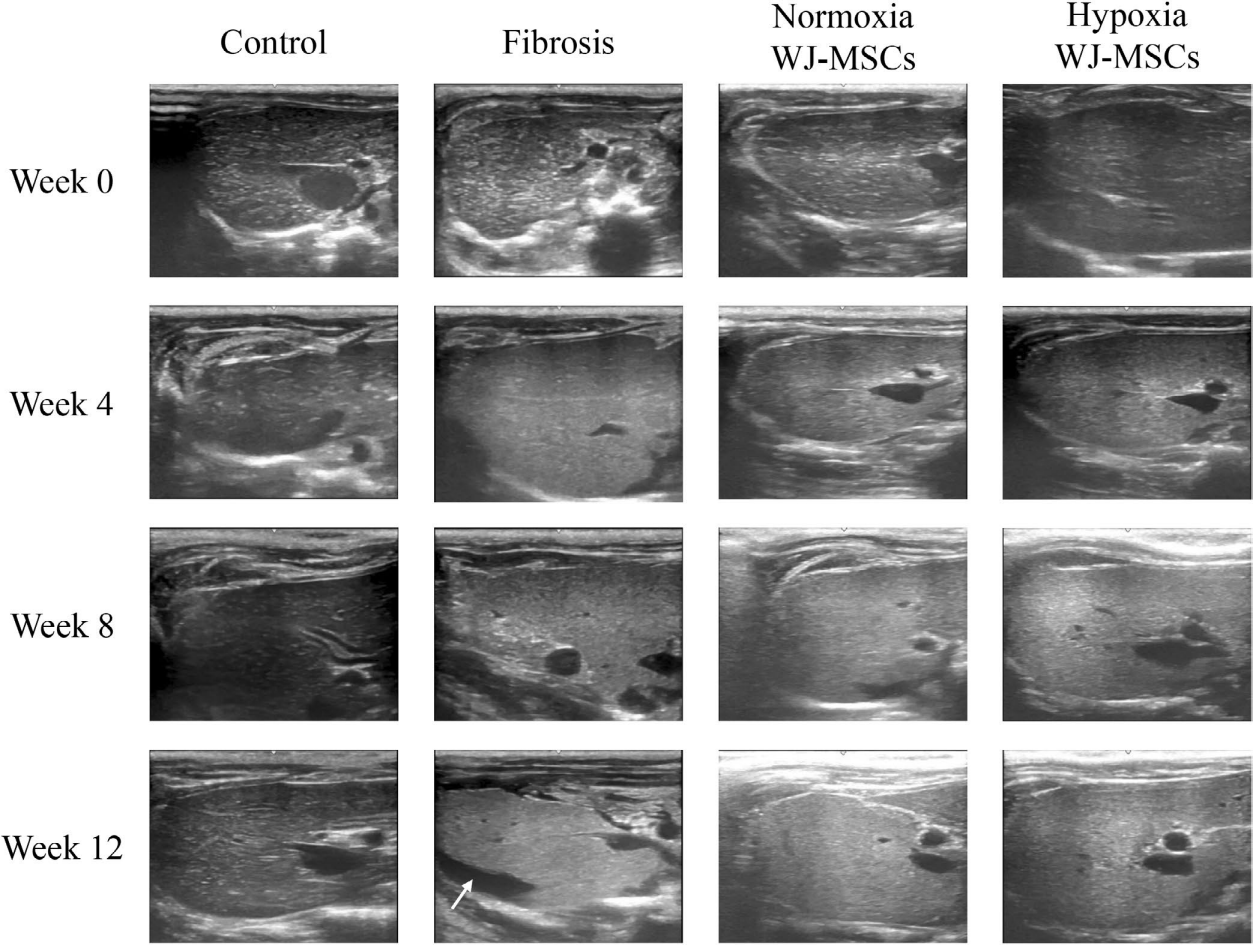
Ultrasound imaging analysis of liver fibrosis progression in rats. Ultrasound imaging was performed for the rat's liver at Week 0, 4, 8, and 12. These images represented the right lobe of the liver of the different groups. The liver of the fibrosis group at Week 8 and Week 12 showed obvious macronodular cirrhotic change of the parenchyma with a mild to moderate amount of ascites (white arrow) while the liver of the normoxia WJ‐MSCs and hypoxia WJ‐MSCs transplantation groups showed only mild macronodular cirrhotic change without ascites.

Ultrasound was performed at Weeks 0, 4, 8, and 12, focusing on both rat's liver lobes, with the right lobe as a representative example. Following CCl_4_‐induced fibrosis, the fibrosis and WJ‐MSC‐treated groups showed increased signal intensity compared to controls. By Week 8, the fibrosis group displayed pronounced macronodular cirrhotic changes, and by Week 12, mild to moderate ascites became evident (white arrow). In contrast, the normoxic and hypoxic WJ‐MSC groups demonstrated reduced ultrasound signal intensity, indicative of only mild cirrhotic changes, with no signs of ascites compared to the fibrosis group (Figure 1).

### 3.2 Serum Biochemical Analysis

**FIGURE 2:**
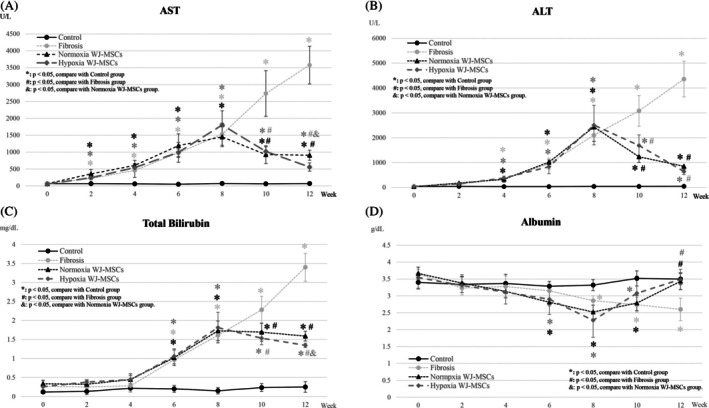
Serum biochemical analysis in rats over a 12‐week period. Blood samples were collected at Week 0, 2, 4, 6, 8, 10, and 12 for serum biochemical analysis. The values of AST (A), ALT (B), total bilirubin (C), and albumin (D) were measured. *p < 0.05, compare with control group. ^#^ *p* < 0.05, compare with fibrosis group. ^&^ *p* < 0.05, compare with normoxia WJ‐MSCs group.

Liver function was assessed by measuring AST, ALT, T‐Bil, and albumin levels over 12 weeks. After a single‐dose (1 × 10^7^ WJ‐MSCs/kg) administration at Week 8, both WJ‐MSC groups showed significant AST and ALT reductions at Weeks 10 and 12 (*p* < 0.05) compared to the fibrosis group, indicating rapid hepatoprotection (Figure 2A,B). Notably, hypoxia WJ‐MSCs exhibited a greater AST reduction at Week 12 (*p* < 0.05) (Figure 2A), while ALT levels showed no significant difference between groups (Figure 2B).

T‐Bil levels were significantly lower in both WJ‐MSC groups at Weeks 8 and 10 (*p* < 0.01), with hypoxia WJ‐MSCs showing a greater reduction at Week 10 (*p* < 0.05) (Figure 2C). Albumin levels remained unchanged at Week 8 but significantly increased in both groups by Week 10 (*p* < 0.05) compared to the fibrosis group (Figure 2D).

### 3.3 PET/MR Imaging and Inflammatory Marker Analysis

**FIGURE 3:**
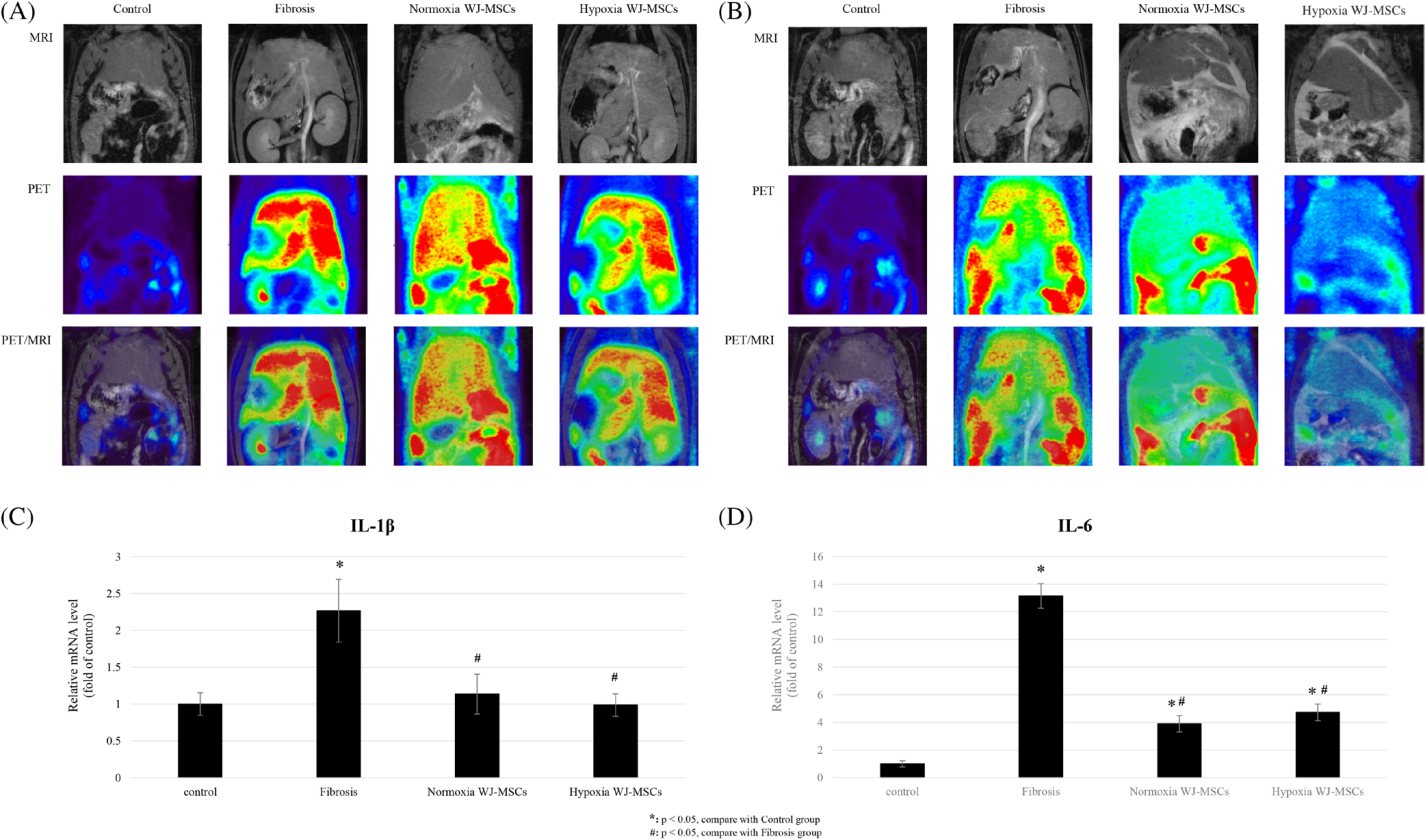
PET/MR imaging and gene expression reveal metabolic and inflammatory changes in rat liver fibrosis. Abdominal PET/MR imaging of rats at Week 8, coronary section (A). Abdominal PET/MR imaging of rats at Week 12, coronary section (B). Quantitative real‐time PCR of the inflammatory gene expression of the rat's liver, including IL‐1β (C) and IL‐6 (D). *p < 0.05, compared with control group. ^#^ *p* < 0.05, compared with fibrosis group. ^&^ *p* < 0.05, compared with normoxia WJ‐MSCs group.

^18^F‐FDG PET/MR imaging assessed metabolic changes. At Week 8, all fibrosis groups showed increased ^18^F‐FDG uptake, indicating heightened metabolic activity and inflammation (Figure 3A). By Week 12, both WJ‐MSC‐treated groups exhibited a significant reduction, suggesting improved metabolic recovery and reduced inflammatory burden. Hypoxia WJ‐MSCs showed a more pronounced decrease, indicating greater therapeutic efficacy in restoring metabolic homeostasis (Figure 3B).

qPCR analysis at Week 12 confirmed significant IL‐1β and IL‐6 downregulation in both WJ‐MSC groups (*p* < 0.05) compared to the fibrosis group. However, no significant difference was observed between hypoxia and normoxia groups (Figure 3C,D).

### 3.4 Histological and Fibrosis Marker Analysis

**FIGURE 4:**
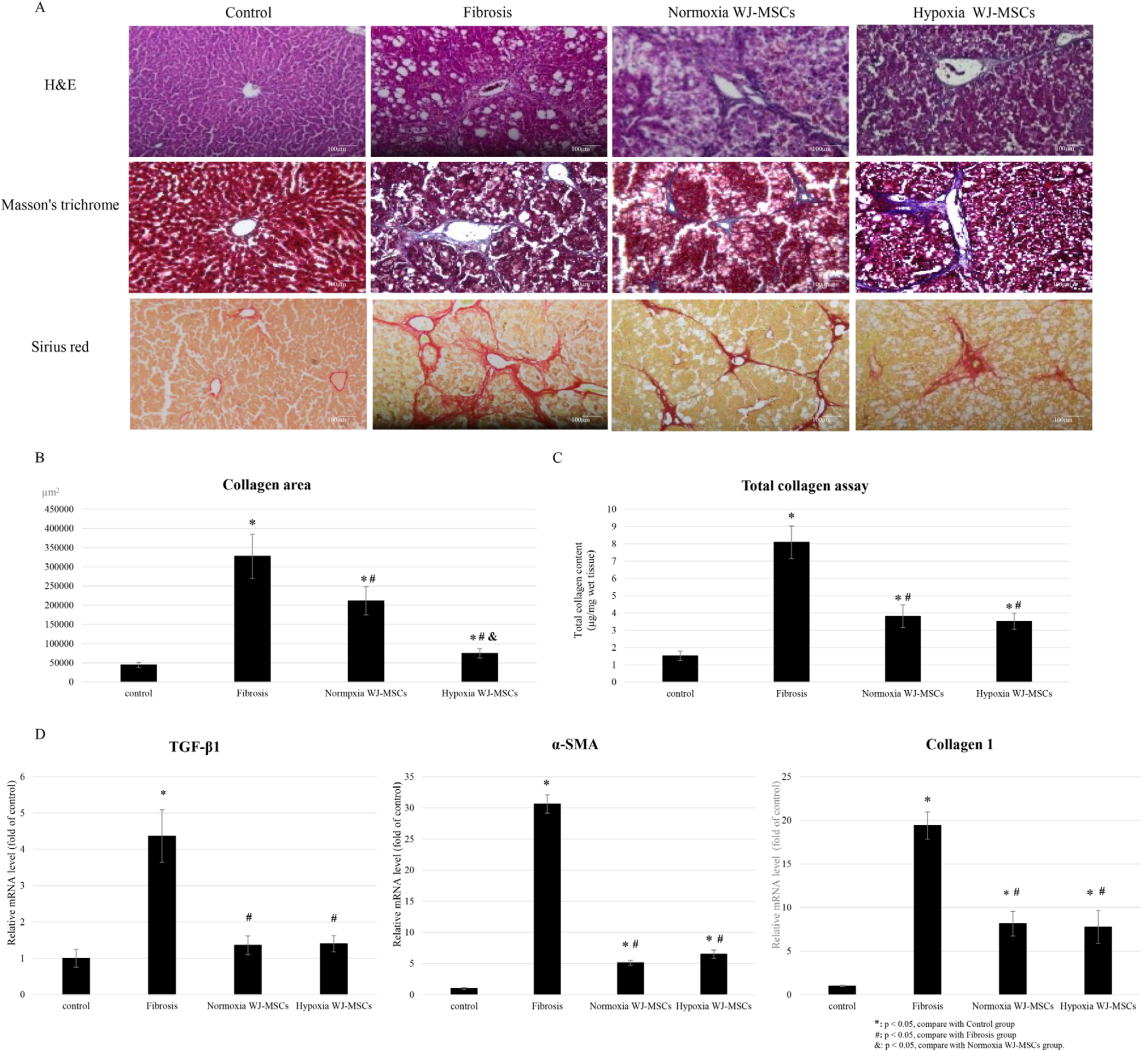
Liver fibrosis morphology, quantification, and MSC effects on fibrosis‐related gene and protein expression. H&E staining, Masson's trichrome staining, and Sirius Red staining of rat liver at Week 12 (A), scale bar = 100 μm. Quantitative analysis of the liver fibrosis area at Week 12 (B). Collagen content analysis of the liver tissue using a collagen assay at Week 12 (C). Quantitative real‐time PCR of the fibrosis‐related gene expression of the rat's liver, including TGF‐β, α‐SMA, and Collagen 1 at Week 12 (D). *p < 0.05, compare with control group. ^#^ *p* < 0.05, compare with fibrosis group. ^&^ *p* < 0.05, compare with normoxia WJ‐MSCs group.

At Week 12, liver tissues were analyzed using H&E, Masson's trichrome, and Sirius Red staining to assess structure and fibrosis. H&E staining showed severe hepatic disruption in the fibrosis group, while Masson's trichrome confirmed reduced fibrotic tissue in both WJ‐MSC‐treated groups. Sirius Red staining, specific for collagen I and III, confirmed significant collagen reduction in both WJ‐MSC groups. Polarized light microscopy showed greater collagen I reduction in the hypoxia WJ‐MSC group compared to the fibrosis and normoxia groups (Figure 4A).

Quantitative fibrosis analysis showed both WJ‐MSC groups had a significantly lower fibrotic area than the fibrosis group (*p* < 0.05). Moreover, the hypoxia WJ‐MSCs showed a greater reduction than normoxia WJ‐MSCs (*p* < 0.05) (Figure 4B).

Total collagen quantification confirmed a significant reduction in both groups, though no significant difference was observed between hypoxia and normoxia WJ‐MSCs (Figure 4C).

Gene expression analysis showed TGF‐β, α‐SMA, and collagen I upregulation in the fibrosis group, while both WJ‐MSC groups exhibited significant downregulation of these markers (*p* < 0.05), with no significant difference between hypoxia and normoxia groups (Figure 4D).

### 3.5 Liver Regeneration Analysis

**FIGURE 5:**
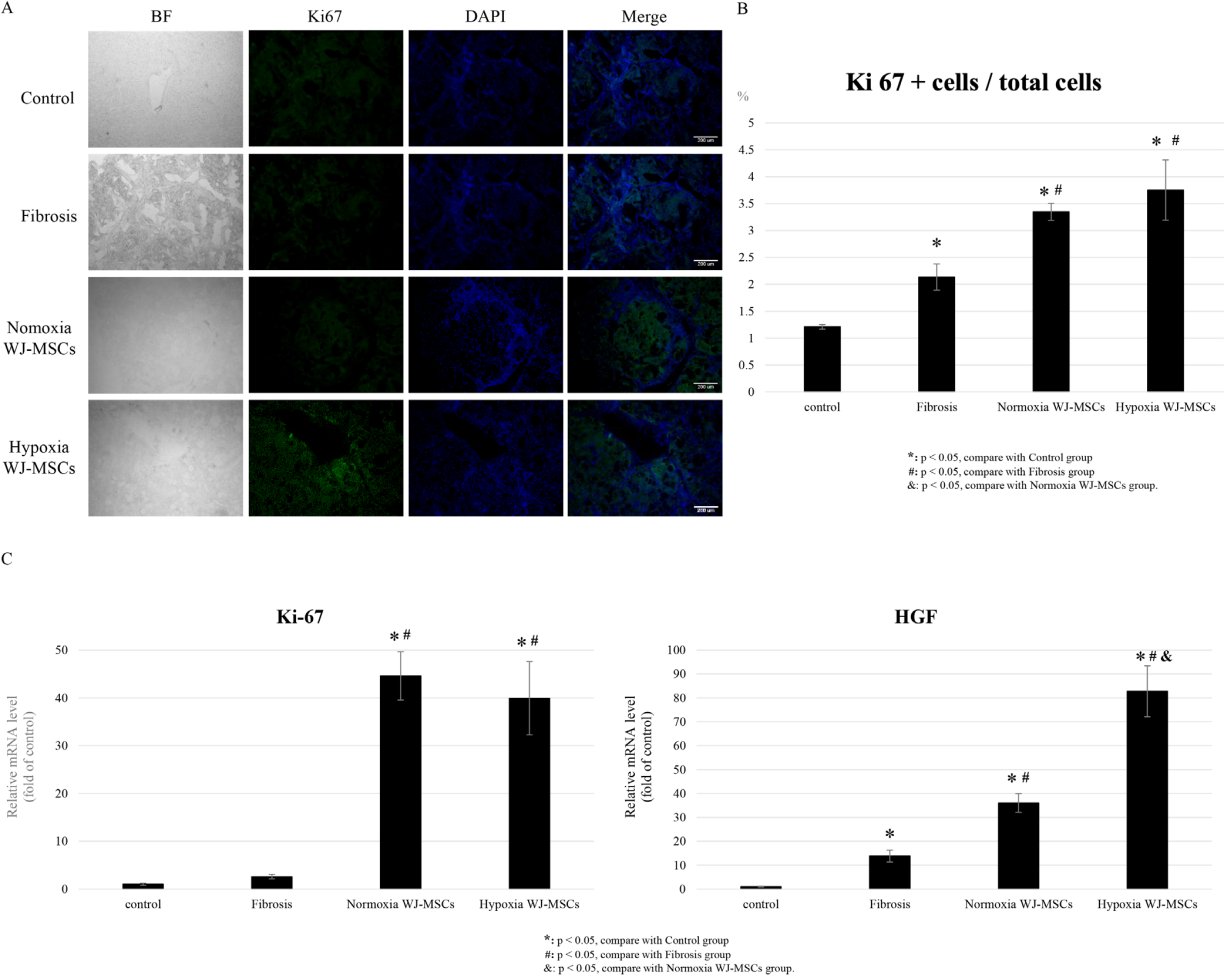
Analysis of liver regeneration markers in rat liver. Immunofluorescence staining of Ki‐67 at Week 12 (A). Scale bar = 200 μm. Quantification of the percentage of the Ki‐67 positive cells in rat liver at Week 12 (B). Quantitative real‐time PCR of the liver regeneration‐related gene expression of the rat's liver, including Ki‐67 and HGF at Week 12 (C). *p < 0.05, compare with control group. ^#^ *p* < 0.05, compare with fibrosis group. ^&^ *p* < 0.05, compare with normoxia WJ‐MSCs group.

Liver regeneration was evaluated using Ki67 immunohistochemistry and qPCR (Figure 5A). Ki67‐positive cells were significantly increased in both WJ‐MSC groups compared to the fibrosis group (*p* < 0.05), with no significant difference between hypoxia and normoxia groups (Figure 5B).

qPCR analysis confirmed upregulated Ki67 expression in both groups, with comparable levels between hypoxia and normoxia WJ‐MSCs (Figure 5C).

HGF expression was significantly higher in both WJ‐MSC groups than in the fibrosis group (*p* < 0.05), with hypoxia WJ‐MSCs showing a greater increase than normoxia WJ‐MSCs (*p* < 0.05) (Figure 5D).

### 3.6 Detection of MSC Presence in Liver and Lung Tissues

**FIGURE 6:**
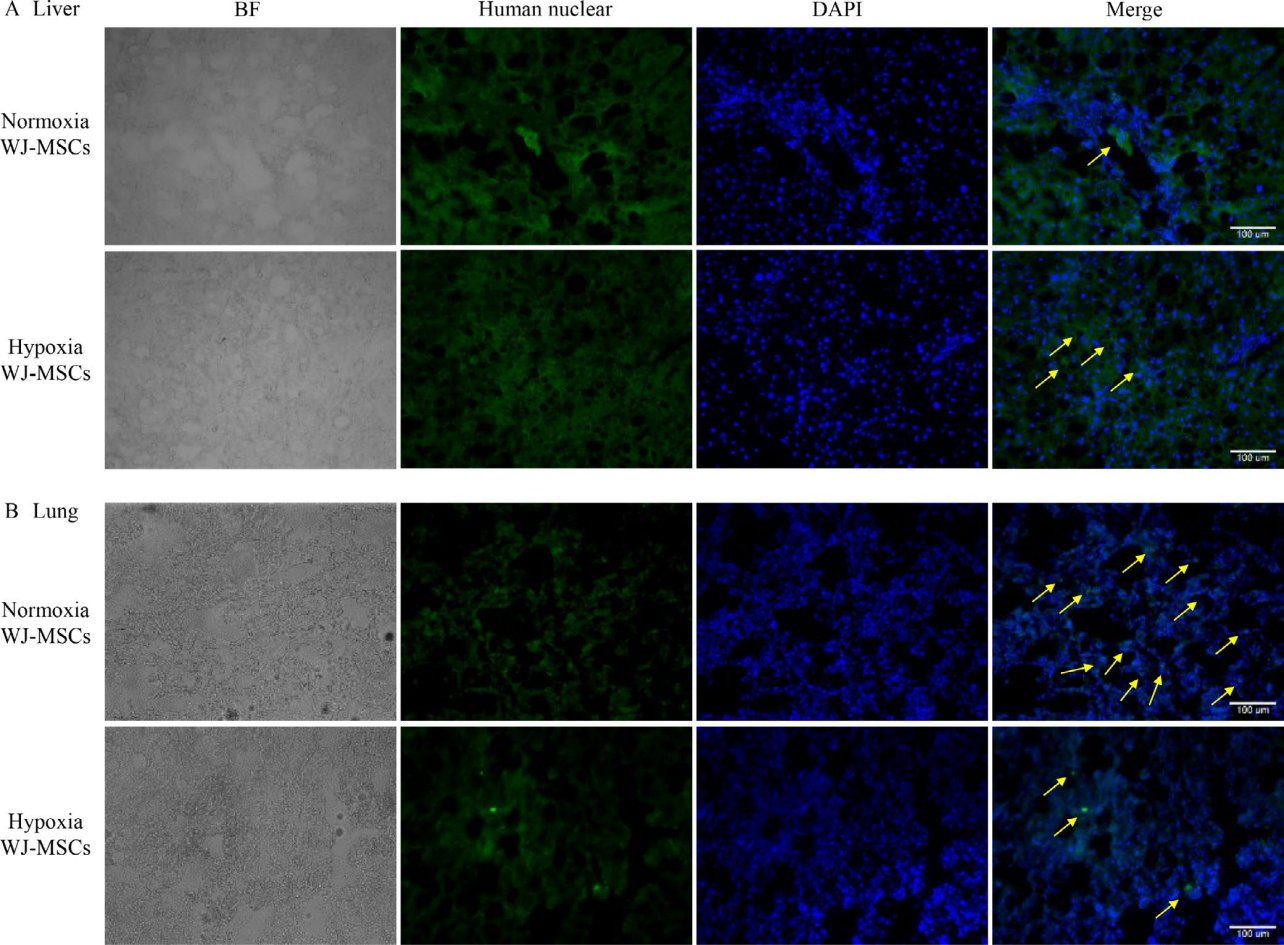
Detection of MSC presence in liver and lung tissues at Week 12. Immunofluorescence staining of human nuclear (yellow arrow) in liver of WJ‐MSCs transplantation group rat at Week 12 (A). Immunofluorescence staining of human nuclear (yellow arrow) in lung of WJ‐MSCs transplantation group rat at Week 12 (B). Scale bar = 100 μm.

To track transplanted WJ‐MSCs, hNA immunohistochemistry was performed on liver and lung tissues at Week 12. The hypoxia WJ‐MSC group exhibited a significantly higher number of hNA‐positive cells in the liver compared to the normoxia group, suggesting improved engraftment and retention (Figure 6A).

In contrast, the normoxia WJ‐MSC group exhibited a higher number of hNA‐positive cells in lung tissue, indicating greater pulmonary entrapment. The hypoxia WJ‐MSC group displayed a lower presence of hNA‐positive cells in the lungs, suggesting enhanced homing and retention in the liver (Figure 6B).

## 4 Discussion

The CCl_4_‐induced liver fibrosis model mimics the progression from acute liver injury to cirrhosis. Unlike previous studies initiating treatment at AST/ALT ~500 U/L [23], this model established late‐stage fibrosis (AST/ALT ~1500/2000 U/L) before WJ‐MSC administration at Week 8, ensuring clinical relevance. To better simulate persistent fibrosis, CCl_4_ exposure continued throughout the study. Peripheral IV injection often leads to MSC entrapment in the lungs, limiting liver delivery [24, 25]. Since 70%–75% of liver blood flow comes from the PV, we used direct PV injection, bypassing the lungs and maximizing liver targeting. Clinically, interventional radiology techniques enable percutaneous PV injection, offering a feasible approach for targeted MSC therapy in liver fibrosis.

Hypoxia preconditioning significantly improved liver function, fibrosis regression, and metabolic recovery compared to normoxia WJ‐MSCs [7, 26]. Biochemical analysis showed greater reductions in AST and T‐Bil levels (*p* < 0.05), indicating enhanced hepatoprotection [22, 27] and bilirubin clearance [6] (Figure 2A,C). While albumin levels were initially unchanged, both WJ‐MSC groups showed significant increases by Week 10, suggesting improved hepatic protein synthesis (Figure 2D) [13]. PET/MR imaging revealed greater metabolic recovery in hypoxia WJ‐MSCs, with a more pronounced reduction in ^18^F‐FDG uptake at Week 12 (Figure 3B), suggesting stronger attenuation of metabolic stress and inflammation [28]. The mechanisms behind this enhanced efficacy likely involve hypoxia‐induced upregulation of HIF‐1α and HIF‐2α, enhancing MSC survival, migration, and paracrine activity. Hypoxia‐exposed MSCs also secrete higher levels of VEGF and HGF, facilitating vascular remodeling, hepatocyte regeneration, and anti‐apoptotic effects [29, 30].

Chronic inflammation drives fibrosis progression, and MSC therapy modulates immune responses [26, 29]. IL‐1β and IL‐6 are key pro‐inflammatory cytokines linked to hepatocyte injury and fibrosis progression. MSCs exert anti‐inflammatory effects by secreting immunomodulatory factors such as IL‐10 and TGF‐β, which suppress inflammatory signaling and promote a shift from a pro‐inflammatory to a reparative microenvironment [19, 22]. qPCR at Week 12 showed significant IL‐1β and IL‐6 downregulation in both WJ‐MSC groups (*p* < 0.05) (Figure 3C,D). However, no significant difference was observed between hypoxia and normoxia WJ‐MSCs, suggesting that immunomodulation is a general MSC effect rather than a specific outcome of hypoxia preconditioning. It is possible that both groups reached a therapeutic threshold for cytokine suppression [18], limiting further improvements. Additionally, hypoxia preconditioning mainly enhances MSC survival, migration, and paracrine secretion, which may not directly impact cytokine suppression [30].

Histological analysis confirmed hypoxia‐preconditioned WJ‐MSCs led to greater fibrosis regression than normoxia WJ‐MSCs. Sirius Red staining revealed significantly lower collagen deposition in both MSC‐treated groups, with hypoxia WJ‐MSCs showing more pronounced reduction under polarized light microscopy (Figure 4A). Quantitative analysis confirmed hypoxia WJ‐MSCs exhibited a significantly lower fibrotic area (*p* < 0.05) (Figure 4B), indicating a stronger antifibrotic response [12]. However, total collagen content analysis showed no significant difference between the two MSC groups (Figure 4C), likely due to measurement limitations, as both functional and degraded collagen were quantified. While hypoxia WJ‐MSCs may have been more effective in breaking down fibrotic collagen, the accumulation of degraded fragments could have masked the differences in total collagen content. The enhanced antifibrotic effects of hypoxia WJ‐MSCs may stem from higher MMP‐2 and MMP‐9 secretion, promoting ECM degradation and HSC inactivation [31]. Although structural improvements were observed, the gene expression levels of TGF‐β, α‐SMA, and collagen I were similarly downregulated in both the hypoxia‐ and normoxia‐treated WJ‐MSC groups (Figure 4D). These results suggest that the antifibrotic effects of WJ‐MSCs may be mediated through paracrine mechanisms such as the secretion of antifibrotic and regenerative factors rather than direct transcriptional suppression of fibrogenic genes. Moreover, the timing of analysis at Week 12 may have coincided with a post‐inflammatory remodeling phase, during which matrix degradation was more prominent than active fibrogenesis. The superior therapeutic effects observed with hypoxia‐preconditioned WJ‐MSCs might be related to enhanced matrix remodeling, potentially through increased MMP‐2 and MMP‐9 activity. Overall, while both WJ‐MSC groups effectively attenuated fibrogenesis, hypoxia preconditioning may further promote tissue remodeling and regeneration beyond the level of gene regulation.

Liver regeneration requires hepatocyte proliferation and microenvironmental support. Ki67 marks cell division, while HGF promotes tissue repair and hepatocyte survival [29, 32, 33]. Both WJ‐MSC groups significantly increased Ki67 and HGF expression (*p* < 0.05) compared to the fibrosis group (Figure 5B–D). However, hypoxia WJ‐MSCs exhibited significantly higher HGF expression (*p* < 0.05) (Figure 5D), while Ki67 levels remained similar (Figure 5C), suggesting enhanced regenerative signaling rather than directly increased hepatocyte proliferation. HGF likely supports long‐term tissue repair, while Ki67 reflects peak hepatocyte proliferation. Since MSC‐driven regeneration relies on paracrine signaling, hypoxia WJ‐MSCs may amplify HGF secretion, improving the regenerative microenvironment without further increasing Ki67 expression. These findings highlight hypoxia WJ‐MSCs as a promising therapy for liver regeneration in fibrosis treatment.

Hypoxia preconditioning enhanced MSC engraftment in the liver while reducing pulmonary entrapment. Immunohistochemistry at Week 12 showed higher liver retention in the hypoxia group, while normoxia MSCs had greater lung sequestration (Figure 6A,B). PV injection improved liver engraftment by bypassing pulmonary circulation, preventing MSC entrapment in the lungs [34]. Beyond delivery route, hypoxia preconditioning enhances homing mechanisms. Hypoxia‐exposed MSCs upregulate CXCR4, which interacts with SDF‐1 in injured liver tissue, promoting migration [35, 36]. Additionally, hypoxia increases adhesion molecule expression, improving MSC attachment to hepatic endothelial cells [37], while greater oxidative stress resistance enhances MSC survival in the inflamed liver microenvironment. These findings suggest that hypoxia preconditioning and PV administration enhance MSC targeting and retention, optimizing their therapeutic potential for liver fibrosis.

Hypoxia preconditioning likely enhances MSC therapeutic effects by activating CXCR4/SDF‐1 signaling, promoting cell migration and retention, and increasing the secretion of factors such as MMP‐2, MMP‐9, and HGF to support fibrosis resolution and liver regeneration. In our study, although these mechanisms were discussed based on functional improvements and previous reports, we did not directly measure HIF‐1α expression, CXCR4 levels, or MMP activity. Future studies will address these mechanistic gaps through protein quantification, secretome profiling, and functional migration assays. Overall, our findings support hypoxia preconditioning as a promising strategy to optimize MSC‐based therapy for liver fibrosis, warranting further clinical investigation.

This study has several limitations. First, the CCl_4_‐induced fibrosis model primarily reflects chemical injury and does not fully represent the chronic fibrogenesis seen in viral hepatitis or steatohepatitis. Although it provides a reproducible platform for evaluating therapeutic effects, it cannot fully capture the complexity of human liver disease. Future studies will apply hypoxia‐preconditioned WJ‐MSCs in diet‐induced NASH models and viral mimetic models to validate their efficacy across different etiologies. Second, the 4‐week follow‐up period captured only early therapeutic outcomes. Given the chronic and progressive nature of liver fibrosis, longer observation is necessary to assess the durability of treatment effects. We plan to extend the follow‐up to 8–12 weeks, incorporating serial imaging, biomarker analyses, and histological assessments to better evaluate sustained liver recovery and MSC persistence. Finally, although single‐dose PV injection was effective, further optimization of dosing regimens and delivery methods is needed to enhance clinical applicability and maximize therapeutic benefits.

In conclusion, hypoxia‐preconditioned WJ‐MSCs highlight superior therapeutic efficacy for liver fibrosis, enhancing liver function, fibrosis regression, and regeneration. They exhibit improved engraftment and stronger anti‐inflammatory effects. These findings support hypoxia preconditioning as a promising strategy to optimize MSC‐based therapy for liver fibrosis, warranting further clinical investigation.

## Ethics Statement

The use of human tissue and laboratory animals in this study was approved by the Institutional Review Board Committee at Taipei Veterans General Hospital, Research Ethics Committee and the Animal Research Committee at National Yang Ming Chiao Tung University (IRB approval no. 2018‐02‐008BC and IACUC approval no. 981242).

## Conflicts of Interest

The authors declare no conflicts of interest.

## Data Availability

Data sharing not applicable to this article as no datasets were generated or analyzed during the current study.

## References

[1] R. Bataller and D. A. Brenner , “Liver Fibrosis,” Journal of Clinical Investigation 115, no. 2 (2005): 209–218. 15690074 10.1172/JCI24282 PMC546435

[2] V. Hernandez‐Gea and S. L. Friedman , “Pathogenesis of Liver Fibrosis,” Annual Review of Pathology: Mechanisms of Disease 6, no. 1 (2011): 425–456. 10.1146/annurev-pathol-011110-130246 21073339

[3] P. Ginès , A. Krag , J. G. Abraldes , E. Solà , N. Fabrellas , and P. S. Kamath , “Liver Cirrhosis,” Lancet 398, no. 10308 (2021): 1359–1376. 34543610 10.1016/S0140-6736(21)01374-X

[4] R. P. Tamayo , “Is Cirrhosis of the Liver Experimentally Produced by Cc14 an Adequate Model of Human Cirrhosis?,” Hepatology 3, no. 1 (1983): 112–120. 6337081 10.1002/hep.1840030118

[5] D. Scholten , J. Trebicka , C. Liedtke , and R. Weiskirchen , “The Carbon Tetrachloride Model in Mice,” Laboratory Animals 49, no. 1 (2015): 4–11. 25835733 10.1177/0023677215571192

[6] J. Jung , J. H. Choi , Y. Lee , et al., “Human Placenta‐Derived Mesenchymal Stem Cells Promote Hepatic Regeneration in CCl_4_‐Injured Rat Liver Model via Increased Autophagic Mechanism,” Stem Cells 31, no. 8 (2013): 1584–1596. 23592412 10.1002/stem.1396

[7] Y. Kojima , A. Tsuchiya , M. Ogawa , et al., “Mesenchymal Stem Cells Cultured Under Hypoxic Conditions Had a Greater Therapeutic Effect on Mice With Liver Cirrhosis Compared to Those Cultured Under Normal Oxygen Conditions,” Regenerative Therapy 11 (2019): 269–281. 31667206 10.1016/j.reth.2019.08.005 PMC6813562

[8] J. M. Slack , “What Is a Stem Cell?,” Wiley Interdisciplinary Reviews: Developmental Biology 7, no. 5 (2018): e323. 29762894 10.1002/wdev.323

[9] Y. Cao , C. Ji , and L. Lu , “Mesenchymal Stem Cell Therapy for Liver Fibrosis/Cirrhosis,” Annals of Translational Medicine 8, no. 8 (2020): 562. 32775363 10.21037/atm.2020.02.119 PMC7347778

[10] H. S. Wang , S. C. Hung , S. T. Peng , et al., “Mesenchymal Stem Cells in the Wharton's Jelly of the Human Umbilical Cord,” Stem Cells 22, no. 7 (2004): 1330–1337. 15579650 10.1634/stemcells.2004-0013

[11] S. Kern , H. Eichler , J. Stoeve , H. Klüter , and K. Bieback , “Comparative Analysis of Mesenchymal Stem Cells From Bone Marrow, Umbilical Cord Blood, or Adipose Tissue,” Stem Cells 24, no. 5 (2006): 1294–1301. 16410387 10.1634/stemcells.2005-0342

[12] K. Chen , D. Wang , W. T. du , et al., “Human Umbilical Cord Mesenchymal Stem Cells hUC‐MSCs Exert Immunosuppressive Activities Through a PGE2‐Dependent Mechanism,” Clinical Immunology 135, no. 3 (2010): 448–458. 20207200 10.1016/j.clim.2010.01.015

[13] L. Zhang , D. Zhou , J. Li , et al., “Effects of Bone Marrow‐Derived Mesenchymal Stem Cells on Hypoxia and the Transforming Growth Factor Beta 1 (TGFβ‐1) and SMADs Pathway in a Mouse Model of Cirrhosis,” Medical Science Monitor: International Medical Journal of Experimental and Clinical Research 25 (2019): 7182–7190. 31550244 10.12659/MSM.916428 PMC6775794

[14] P. C. Tsai , T. W. Fu , Y. M. A. Chen , et al., “The Therapeutic Potential of Human Umbilical Mesenchymal Stem Cells From Wharton's Jelly in the Treatment of Rat Liver Fibrosis,” Liver Transplantation 15, no. 5 (2009): 484–495. 19399744 10.1002/lt.21715

[15] J. Yu , S. Yin , W. Zhang , et al., “Hypoxia Preconditioned Bone Marrow Mesenchymal Stem Cells Promote Liver Regeneration in a Rat Massive Hepatectomy Model,” Stem Cell Research & Therapy 4 (2013): 1–9. 23856418 10.1186/scrt234 PMC3854783

[16] S. C. Lee , H. J. Jeong , S. K. Lee , and S. J. Kim , “Hypoxic Conditioned Medium From Human Adipose‐Derived Stem Cells Promotes Mouse Liver Regeneration Through JAK/STAT3 Signaling,” Stem Cells Translational Medicine 5, no. 6 (2016): 816–825. 27102647 10.5966/sctm.2015-0191 PMC4878330

[17] N. V. Iyer , L. E. Kotch , F. Agani , et al., “Cellular and Developmental Control of O_2_ Homeostasis by Hypoxia‐Inducible Factor 1α,” Genes & Development 12, no. 2 (1998): 149–162. 9436976 10.1101/gad.12.2.149 PMC316445

[18] Y.‐W. Lan , K. B. Choo , C. M. Chen , et al., “Hypoxia‐Preconditioned Mesenchymal Stem Cells Attenuate Bleomycin‐Induced Pulmonary Fibrosis,” Stem Cell Research & Therapy 6 (2015): 1–17. 25986930 10.1186/s13287-015-0081-6 PMC4487587

[19] N. Takizawa , N. Okubo , M. Kamo , et al., “Bone Marrow‐Derived Mesenchymal Stem Cells Propagate Immunosuppressive/Anti‐Inflammatory Macrophages in Cell‐to‐Cell Contact‐Independent And‐Dependent Manners Under Hypoxic Culture,” Experimental Cell Research 358, no. 2 (2017): 411–420. 28712928 10.1016/j.yexcr.2017.07.014

[20] A. Garg and P. N. Newsome , “Bone Marrow Mesenchymal Stem Cells and Liver Regeneration: Believe the Hypoxia!,” Stem Cell Research & Therapy 4 (2013): 1–2. 24004909 10.1186/scrt319 PMC4056659

[21] P. Prasajak and W. Leeanansaksiri , “Developing a New Two‐Step Protocol to Generate Functional Hepatocytes From Wharton's Jelly‐Derived Mesenchymal Stem Cells Under Hypoxic Condition,” Stem Cells International 2013, no. 1 (2013): 762196. 23818908 10.1155/2013/762196 PMC3683497

[22] H. Li , X. Q. Ji , S. M. Zhang , and R. H. Bi , “Hypoxia and Inflammatory Factor Preconditioning Enhances the Immunosuppressive Properties of Human Umbilical Cord Mesenchymal Stem Cells,” World Journal of Stem Cells 15, no. 11 (2023): 999–1016. 38058960 10.4252/wjsc.v15.i11.999 PMC10696190

[23] X. Rong , J. Liu , X. Yao , T. Jiang , Y. Wang , and F. Xie , “Human Bone Marrow Mesenchymal Stem Cells‐Derived Exosomes Alleviate Liver Fibrosis Through the Wnt/β‐Catenin Pathway,” Stem Cell Research & Therapy 10 (2019): 1–11. 30885249 10.1186/s13287-019-1204-2 PMC6421647

[24] A. Kurtz , “Mesenchymal Stem Cell Delivery Routes and Fate,” International Journal of Stem Cells 1, no. 1 (2008): 1–7. 24855503 10.15283/ijsc.2008.1.1.1 PMC4021770

[25] L. L. Bagno , A. G. Salerno , W. Balkan , and J. M. Hare , “Mechanism of Action of Mesenchymal Stem Cells (MSCs): Impact of Delivery Method,” Expert Opinion on Biological Therapy 22, no. 4 (2022): 449–463. 34882517 10.1080/14712598.2022.2016695 PMC8934282

[26] Y. W. Eom , K. Y. Shim , and S. K. Baik , “Mesenchymal Stem Cell Therapy for Liver Fibrosis,” Korean Journal of Internal Medicine 30, no. 5 (2015): 580–589. 26354051 10.3904/kjim.2015.30.5.580 PMC4578027

[27] C. Hu , L. Zhao , J. Duan , and L. Li , “Strategies to Improve the Efficiency of Mesenchymal Stem Cell Transplantation for Reversal of Liver Fibrosis,” Journal of Cellular and Molecular Medicine 23, no. 3 (2019): 1657–1670. 30635966 10.1111/jcmm.14115 PMC6378173

[28] I. Rosova and J. A. Nolta , “Hypoxic Preconditioning Results in Increased Motility and Improved Therapeutic Potential of Human Mesenchymal Stem Cells in a Xenograft Hind Limb Ischemia Injury Model,” Blood 110, no. 11 (2007): 217. 10.1634/stemcells.2007-1104 PMC3017477 18511601

[29] K. Hoffmann , A. J. Nagel , K. Tanabe , et al., “Markers of Liver Regeneration—The Role of Growth Factors and Cytokines: A Systematic Review,” BMC Surgery 20 (2020): 1–15. 32050952 10.1186/s12893-019-0664-8 PMC7017496

[30] Q. Wang , Y. Li , H. Yuan , et al., “Hypoxia Preconditioning of Human Amniotic Mesenchymal Stem Cells Enhances Proliferation and Migration and Promotes Their Homing via the HGF/C‐MET Signaling Axis to Augment the Repair of Acute Liver Failure,” Tissue and Cell 87 (2024): 102326. 38442547 10.1016/j.tice.2024.102326

[31] Y. P. Han , “Matrix Metalloproteinases, the Pros and Cons, in Liver Fibrosis,” Journal of Gastroenterology and Hepatology 21 (2006): S88–S91. 16958682 10.1111/j.1440-1746.2006.04586.x PMC2646497

[32] H. Gilgenkrantz and A. C. de l'Hortet , “Understanding Liver Regeneration: From Mechanisms to Regenerative Medicine,” American Journal of Pathology 188, no. 6 (2018): 1316–1327. 29673755 10.1016/j.ajpath.2018.03.008

[33] G. K. Michalopoulos and B. Bhushan , “Liver Regeneration: Biological and Pathological Mechanisms and Implications,” Nature Reviews Gastroenterology & Hepatology 18, no. 1 (2021): 40–55. 32764740 10.1038/s41575-020-0342-4

[34] C. H. Masterson , A. Tabuchi , G. Hogan , et al., “Intra‐Vital Imaging of Mesenchymal Stromal Cell Kinetics in the Pulmonary Vasculature During Infection,” Scientific Reports 11, no. 1 (2021): 5265. 33664277 10.1038/s41598-021-83894-7 PMC7933415

[35] C. Cencioni , M. C. Capogrossi , and M. Napolitano , “The SDF‐1/CXCR4 Axis in Stem Cell Preconditioning,” Cardiovascular Research 94, no. 3 (2012): 400–407. 22451511 10.1093/cvr/cvs132

[36] H. Liu , S. Liu , Y. Li , et al., “The Role of SDF‐1‐CXCR4/CXCR7 Axis in the Therapeutic Effects of Hypoxia‐Preconditioned Mesenchymal Stem Cells for Renal Ischemia/Reperfusion Injury,” PLoS One 7, no. 4 (2012): e34608. 22511954 10.1371/journal.pone.0034608 PMC3325280

[37] I. V. Kholodenko , R. V. Kholodenko , and K. N. Yarygin , “The Crosstalk Between Mesenchymal Stromal/Stem Cells and Hepatocytes in Homeostasis and Under Stress,” International Journal of Molecular Sciences 24, no. 20 (2023): 15212. 37894893 10.3390/ijms242015212 PMC10607347

